# Antimicrobial Resistance in Commensal *Escherichia coli* Isolated from Pigs and Pork Derived from Farms Either Routinely Using or Not Using In-Feed Antimicrobials

**DOI:** 10.1089/mdr.2018.0154

**Published:** 2018-09-10

**Authors:** Kittitat Lugsomya, Jitrapa Yindee, Waree Niyomtham, Chanwit Tribuddharat, Padet Tummaruk, David J. Hampson, Nuvee Prapasarakul

**Affiliations:** ^1^ Department of Veterinary Microbiology, Faculty of Veterinary Science, Chulalongkorn University, Bangkok, Thailand.; ^2^ Department of Microbiology, Faculty of Medicine Siriraj Hospital, Mahidol University, Bangkok, Thailand.; ^3^ Department of Obstetrics Gynaecology and Reproduction, Faculty of Veterinary Science, Chulalongkorn University, Bangkok, Thailand.; ^4^ School of Veterinary and Life Sciences, Murdoch University, Perth, Australia.; ^5^ College of Veterinary and Life Sciences, City University of Hong Kong, Kowloon Tong, Hong Kong SARS.; ^6^ Diagnosis and Monitoring of Animal Pathogens Research Unit, Chulalongkorn University, Bangkok, Thailand.

**Keywords:** antimicrobial resistance, *Escherichia coli*, extended-spectrum beta-lactamese-producing *Escherichia coli*, longitudinal study, pig production, pork

## Abstract

The aims of this study were (i) to evaluate whether routine in-feed antimicrobial use in pigs or not resulted in differences in antimicrobial resistance (AMR) *E. coli* at different pig producing stages, and (ii) to determine whether resistant strains were presented in pig meat postslaughter. A total of 300 commensal *E. coli* isolates were obtained and examined for antibiograms, AMR genes, plasmid replicons, and molecular types. The isolates were from two farms either using (A) or not using in-feed antimicrobials (NA), sampled four times during the production cycle and once postslaughter. *E. coli* resistant to aminoglycosides containing *aadA1, aadA2*, and *aadB* and extended-spectrum beta-lactamase-producing (ESBLP) *E. coli* containing *bla*_CTX-M-1_ were significantly increased in the nursery and growing periods in farm A compared to farm NA. IncI1-Iγ and IncHI2 were common in the nursery period and were shown to transfer *bla*_CTX-M_ genes by conjugation. ST10 was the most common type only found in live pigs. ST604, ST877, ST1209, and ST2798 ESBLP were found only in live pigs, whereas ST72, ST302, and ST402 ESBLP were found in pig meat.

## Introduction

The ongoing increase in antimicrobial resistance (AMR) in enteric bacteria in production animals and their potential transmission to humans represent a major threat to public health.^1^ Commensal enteric bacteria such as *Escherichia coli* (*E. coli*), which reside for prolonged periods in the intestinal tract, potentially represent an important reservoir of AMR in the food chain, and moreover they make good representative markers for investigating dynamic changes in AMR genes.^2^ Even though the source of AMR has not always been identified using molecular epidemiological analysis or DNA-based data, bacteria from livestock are believed to be a major source of AMR in the environment, and resistant bacteria and resistance genes may be acquired by the human gut microbiome.^3^

Some contract pig-rearing farms in Thailand routinely use antimicrobials under veterinary prescription as feed additives for prophylaxis against bacterial infections and/or as growth promoters. This use is problematic as it is increasingly understood that imprudent application of antimicrobials during the production cycle may increase the occurrence of AMR bacteria on farms, especially during the immediate postweaning “nursery” period.^4^ To date, most studies have only involved cross-sectional observations taken at specific periods of production, with the studied farms having a lack of availability of historical data about antimicrobial use.^5^ A more holistic understanding should result from longitudinal surveillance at different points through the production cycle to meat at slaughter, especially if exposure or lack of exposure to antimicrobials can be recorded. Such studies should indicate the likelihood of transmission of AMR bacterial from animals on the farm to the product, and hence to the consumer.^5^

The aims of this study were (i) to look for alterations in AMR and characteristics of commensal *E. coli* isolated from cohorts of pigs sampled at different phases during the production cycle on farms, which either were routinely using or not using in-feed antimicrobials, and (ii) to look for similarities between *E. coli* contaminating pig meat and those recovered from the same pigs on the two farms.

## Materials and Methods

### Farms

The study was undertaken at two multisite industrial pig farms from Nakhon Pathom and Chainart Provinces, respectively, both of which were run following the Thai standard livestock farm criteria under the guidance of the Department of Livestock Development. These were designated farm A (using antibiotics) and farm NA (not using antibiotics). In farm A in Nakhon Pathom, a combination of tiamulin and amoxicillin at 100 ppm and 250 ppm, respectively, were routinely mixed into the feed during the postweaning nursery and growing periods. In farm NA located in Chainart, antimicrobials had not been used as a feed additive for over 10 years. Enrofloxacin injections were used in the case of symptomatic bacterial infections in the preweaning period; however; all such treated pigs were excluded from this study. Both farms had over 1,000 sows, had no pig replacement from outside sources, used an all-in all-out production system, and had consistent management for at least 2 years in terms of antimicrobial use and sanitary and biosecurity measures taken. The farms were well managed and kept good records of production and antimicrobial use. On farm A, antimicrobial use was withdrawn at least 30 days before the pigs were slaughtered.

### Animal selection and timing of sampling

For each farm, one healthy pig from each of ten different litters was selected for use in the experiment (a total 10 pigs per group). Each pig was ear tagged and sampled at five periods up to and including postslaughter: the neonatal period (at 5 days of age); the postweaning nursery period (at 8 weeks of age); the growing period (at 14 weeks of age); the finishing period (at 24 weeks of age, just before transport to the abattoir); and following slaughter at the abattoir. None of the pigs showed signs of ill health as judged by weekly routine clinical inspection by veterinarians and the farmer's daily observations, nor did they receive therapeutic antimicrobials during the production cycle. The pigs from the two farms were killed at two different abattoirs.

### Sample collection and bacterial identification

The sampling protocol was approved by the Chulalongkorn University Animal Care and Use Committee (permit number 58/2558). For the pigs in the neonatal and nursery periods, rectal swabs were taken directly and kept in Clary-Blair transport medium. For pigs in the growing and finishing periods, at least 25 g of rectal feces were collected into sterile plastic containers. For the pigs at the abattoir, meat (“pork”) was sampled and placed into sterile containers. Following routine abattoir processing by abattoir staff, half carcasses were suspended on hooks before being moved for subsequent retail sale. A sterile scalpel blade was used to excise at least 25 g of meat from the biceps femoris muscle from a cross-section of the previously opened thigh area of each hanging half-carcass. All samples were delivered to the laboratory at 4°C within 24 hours. The rectal swabs were suspended in 0.85% sodium chloride solution (NSS) and directly spread on Eosin Methylene Blue (EMB) (Oxoid) agar.^6^ For rectal feces, at least 5 g was diluted 10-fold to 10^−4^ and the solutions plated to EMB agar. One gram of each meat sample was placed in a sterile plastic bag with 9 mL of sterile normal saline and blended in a stomacher (Interscience Malaysia) for 3 minutes. After vigorously shaking, 100 μL of the suspension was spread on EMB agar.^7^ All plates were incubated overnight at 37°C. For each sampling time for each animal, three representative colonies from the highest dilution plate were selected for further characterization. Colonies presenting a metallic sheen on EMB plates were selected and confirmed as *E. coli* by their IMViC biochemical reactions, comprising an indole test.^8^

### Antibiogram and extended-spectrum beta-lactamase phenotype confirmation

The basic procedures followed have been described previously.^9^ The minimal inhibitory concentration of antimicrobials for *E. coli* were determined using the AST-GN 38 test kit in the Vitek2 compact automated susceptibility level detection apparatus (BioMérieux, France). The 18 antimicrobials or combination of antimicrobials used were as follows: amikacin (AK: 2–64 μg/mL), amoxicillin (AMX: 2–32 μg/mL), amoxicillin/clavulanic acid (AMC: 2/1-32/16 μg/mL), ampicillin (AMP: 2–32 μg/mL), cefalexin (CEX: 4–64 μg/mL), cefpirome (CPR: 2–16 μg/mL), cefpodoxime (CPD: 0.25–8 μg/mL), ceftiofur (XNL: 1–8 μg/mL), chloramphenicol (C: 2–64 μg/mL), enrofloxacin (ENR: 0.125–4 μg/mL), gentamicin (GEN: 2–64 μg/mL), imipenem (IMP: 1–16 μg/mL), marbofloxacin (MBRL: 0.5–4 μg/mL), nitrofurantoin (NIT: 16–512 μg/mL), piperacillin (PIP: 4–128 μg/mL), tetracycline (TET: 1–16 μg/mL), tobramycin (TOB: 4–16 μg/mL), and trimethoprim/sulfamethoxazole (SXT: 1/19-16/304 μg/mL). *E. coli* ATCC 25922 was used as the control strain. The interpretation of the susceptibility levels for AMP, CPD, CPR, XNL, GEN, ENR, MBR, and TET was performed following the Clinical Laboratory Standards (CLSI) for antimicrobial disks and testing for bacteria isolated from animals (VET2-0S3),^10^ and interpretation for AMX, PIP, AMC, CEX, IMP, AK, TOB, NIT, and SXT was followed according to the CLSI standards for antimicrobial susceptibility testing (M100-S25).^11^

### Extended-spectrum beta-lactamase phenotypic screening and confirmatory test

Extended-spectrum beta-lactamase-producing (ESBLP) *E. coli* were identified using the Vitek2 machine (BioMérieux, France)^12^ and the results were confirmed by the combination disk test following CLSI standards recommendations.^11^ The *bla*_CTX-M_ genes comprising variants *bla*_CTX-M-1_, *bla*_CTX-M-2,_ *bla*_CTX-M-8,_ *bla*_CTX-M-9_, and *bla*_CTX-M-25/26_ were detected by multiplex PCR in all ESBLP strains.^13^ The identity of representative PCR amplicons was confirmed by DNA sequencing and analyzed using Mega 7.0,^14^ with comparisons made to the GenBank database.

To confirm whether *bla*_CTX-M_ genes were located on transmissible plasmids, a conjugation assay was performed using the broth mating technique, as previous described.^15^ The three representative selected donor clones, *E. coli* PCU12-4 (positive for *bla*_CTX-M-1_ group with a single IncI1-Iγ replicon), *E. coli* PCU12-5 (positive for *bla*_CTX-M-1_ group with a single IncHI2 replicon), and PCU12-6 (positive for *bla*_CTX-M-9_ group with a single IncHI2 replicon), were selected. The recipient strain *E. coli* J53 was resistant to sodium azide (Azi^r^) and susceptible to cefotaxime. The transconjugants were selected on Luria-Bertani (LB) agar (Oxoid) supplemented with cefotaxime (2 μg mL^−1^) and sodium azide (100 μg mL^−1^) (Oxoid). Testing for antimicrobial susceptibility, ESBL confirmatory phenotype, PCR detection, and DNA sequencing of *bla*_CTX-M_ genes was performed on the transconjugants, as previously described.^15^

## Detection of genes encoding AMR

DNA was extracted from the bacteria using a Wizard^®^ Genomic DNA Purification Kit (Promega, Germany). Sixteen pairs of primers that were specific for resistance genes in bacteria in the superfamily Enterobacteriaceae were generated (First Base Laboratories, Malaysia), and the PCR thermal cycling conditions used followed previous recommendations. The resistance genes analyzed included *bla*_TEM_ and *bla*_PSE-1_ for ampicillin, amoxicillin, and piperacillin resistance, *aadA1* and *aadA2* for streptomycin resistance, *aadB* for tobramycin and gentamicin resistance, *tet*(A) and *tet*(B) for tetracycline resistance, *sul1*, *sul2*, and *sul3* for sulfonamide resistance, *dfrA1*, *dfrA10*, and *dfrA12* for trimethoprim resistance, and *catA*, *catB*, and *cmlA* for chloramphenicol resistance.^16^ The *bla*_CTX-M-1_ group, *bla*_CTX-M-2_ group, *bla*_CTX-M-8_ group, *bla*_CTX-M-9_ group, and *bla*_CTX-M-25,26_ group were included as these are the most prevalent ESBLP encoding genes.^13^ A representative positive PCR amplicon for each gene was submitted for DNA sequencing and was analyzed by MEGA 7.0.^14^

## Plasmid Replicon Characterization

The Enterobacteriaceae plasmid replicons IncF (IncFIA, IncFIB, IncFIC, and IncFrep), IncI1-Iγ, IncN, IncP, IncW, IncHI1, IncHI2, IncL/M, IncT, IncA/C, IncK, IncB/O, IncX, and IncY were detected using five multiplex and three simplex PCR tests. The primers, PCR conditions, and thermal cycles were as previously described.^17^ Representative positive PCR amplicons for each replicon were submitted for DNA sequencing and analyzed as described above.

## Pulsed-Field Gel Electrophoresis

Pulsed-field gel electrophoresis (PFGE) was performed on the 300 *E. coli* isolates following the CDC standard protocol.^18^ Briefly, *E. coli* DNA in agar plugs was digested with restriction enzyme *Xba*I (Sibenzyme, Russia). Gel electrophoresis was undertaken in a 200 V field at 120° for 18–19 hours, incorporating *Salmonella* serovar Braenderup H9812 DNA as a standard. Dendrograms were generated using the GeneTool program (Syngene, India) and analyzed with the GeneDirectory program (Syngene, India).

## Multilocus Sequence Typing

The sequence types (ST) of the 300 *E. coli* isolates were obtained based on the allelic profiles of seven genes with “housekeeping” function.^19^ STs were obtained using high-throughput multilocus sequence typing (HiMLST) (Boers *et al.*, 2012).^20^ The target genes were amplified by a two-step PCR using a primer sequence for the target genes that included a universal tail sequence primer and an isolate-specific multiplex identifier sequence primer with 454 sequencing-specific nucleotides at the 5′ end. The PCR products were pooled, clonally amplified by emulsion PCR (emPCR) (Roche, Switzerland), and sequenced using the GS junior (Roche). Allele and sequence types (STs) were assigned by the publicly accessible *E. coli* MLST database at http://mlst.warwick.ac.uk/mlst/dbs/Ecoli.

## Data Analysis

Within each farm, comparisons between the rates of the categorical variables (resistance; replicon detection; and resistance gene profiles) of the *E. coli* isolates were made between successive sampling times (i.e., results from the 30 isolates from the neonatal period compared to the 30 from the postweaning period and the 30 from the postweaning period compared to the 30 from the grower period). Between-farm comparisons were made for the 30 isolates at each sampling time for each variable. Results were analyzed using the chi-square test in SPSS version 17.0 (IBM, Armonk, NY). Isolates that were resistant to at least three antimicrobial groups were defined as being multidrug resistant (MDR). The molecular strain types (PFGE profiles and STs) were individually reported by descriptive analysis. The Shannon diversity index (*H*’) was performed to determine the genetic diversity of the *E. coli* strains between farms and was calculated according to the following formula:

\begin{align*}
H^{\prime} = -  \mathop \sum \limits_{i = 0}^s {p_i}{ \rm{ln}}{p_i}
\end{align*}

S was the number of unique genotype; and p_i_ was the number of isolates sharing the same PFGE profiles [i] over the total number of isolates.^21^

## Results

### Phenotypic resistance characterizations and extended-spectrum beta-lactamase phenotype confirmation

**FIG. 1.**
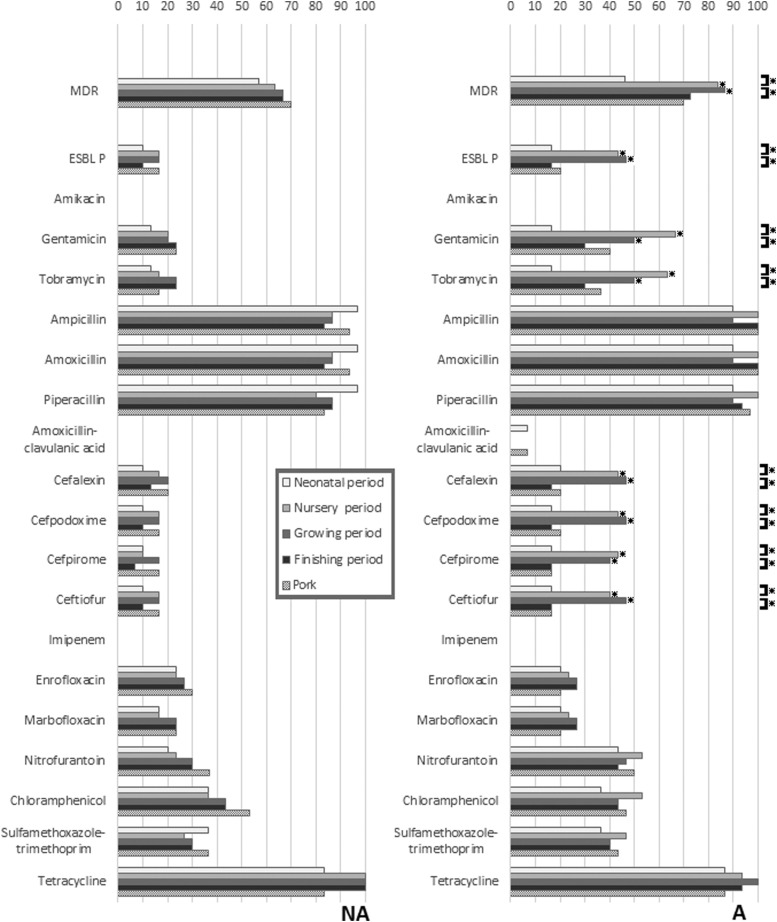
Differences in resistance rates to 18 antimicrobials and ESBLP in *Escherichia coli* isolated from pigs in different periods of the production cycle and postslaughter (pig meat), with comparisons between isolates from the farm using antibiotics (A) and the farm not using antibiotics (NA). *Indicates a significant difference (*p* < 0.05) using chi-squared analysis (*p* < 0.05). Comparisons are made between farms at each sampling period, and within farms at successive sampling periods. ESBLP, extended-spectrum beta-lactamase producing.

A total of 300 commensal *E. coli* isolates were analyzed (3 per pig at each sampling period, 150 per farm). The AMR profiles for the 300 isolates against 18 antimicrobials and their ESBLP profile are shown in Fig. 1. Resistance to nearly all the antimicrobials was common. All or nearly all isolates at all sampling periods (including in pork) from pigs in both farms were resistant to the β-lactam group antibiotics (ampicillin, amoxicillin, and piperacillin), and to tetracycline. Statistically significant differences in resistance rates and ESBLP phenotype between sampling periods were only found on farm A, and only for a few antimicrobials (gentamicin, tobramycin, cefalexin, cefpodoxime, cefpirome, ceftiofur, and ESBLP *E. coli*), with, in each case, the highest rates being in the nursery and grower periods. These same resistances were also statistically significantly higher in the nursery and grower periods in the pigs from farm A compared to those from farm NA. Isolates with MDR phenotypes were common in both farm A (73.3%) and farm NA (64.7%). The most common AMR pattern, AMP-AMX-PIP-TET, was detected at 18.0% and 14.7% for the isolates from farms NA and A, respectively.

### Genotypic resistance characterizations

**FIG. 2.**
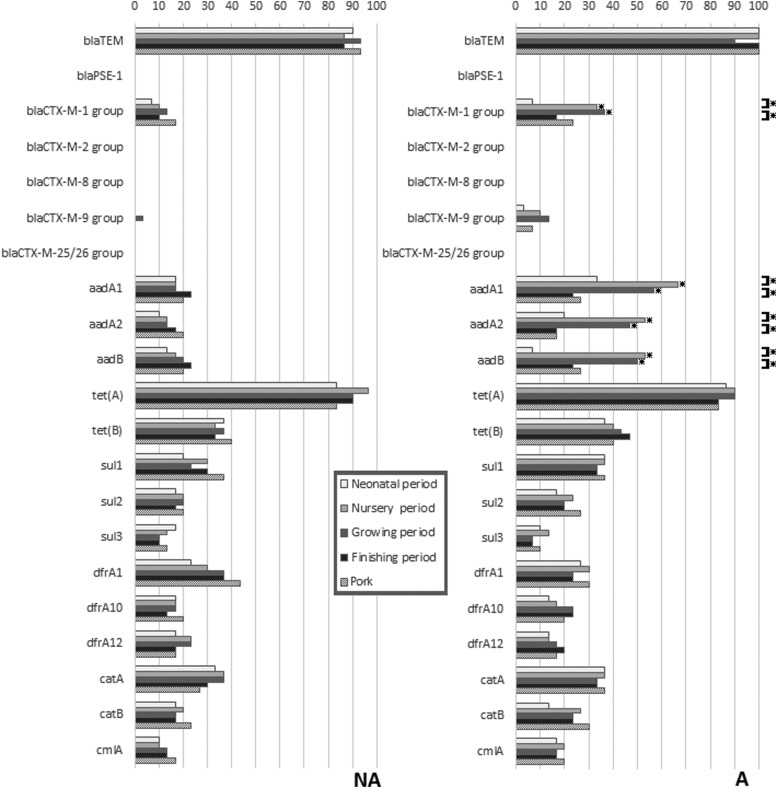
Differences in 20 antimicrobial resistance genes in *E. coli* from pigs in different periods of the production cycle and postslaughter (pig meat), with comparisons between the farm using in-feed antibiotics (A) and the farm not using antibiotics (NA). *Indicates a significant difference (*p* < 0.05) using chi-squared analysis (*p* < 0.05). Comparisons are made between farms at each sampling period, and within farms at successive sampling periods.

The occurrence of 21 genes linked to AMR in the 300 *E. coli* isolates is depicted in Fig. 2. All, but four of the resistance genes were found in some of the isolates. The most common combined resistance gene pattern [*bla*_TEM_ and *tet*(A)] was found in both the NA (18.7%) and the A farm (14.0%). Most AMR genes occurred in less than half the isolates at all sampling periods, except for *bla*_TEM_ and *tet*(A) that were found among nearly all *E. coli* from both farms. Statistically significant differences in rates at different sampling times were only found in farm A, and only for the *bla*_CTX-M-1_ group, *aadA1*, *aadA2*, and *aadB*, where rates were highest in the nursery and growing periods. Statistically significant differences in rates between farms were only found for these same four genes, and only in the nursery and grower periods, where rates were higher in farm A.

### Replicon type detection and conjugation experiment

**FIG. 3.**
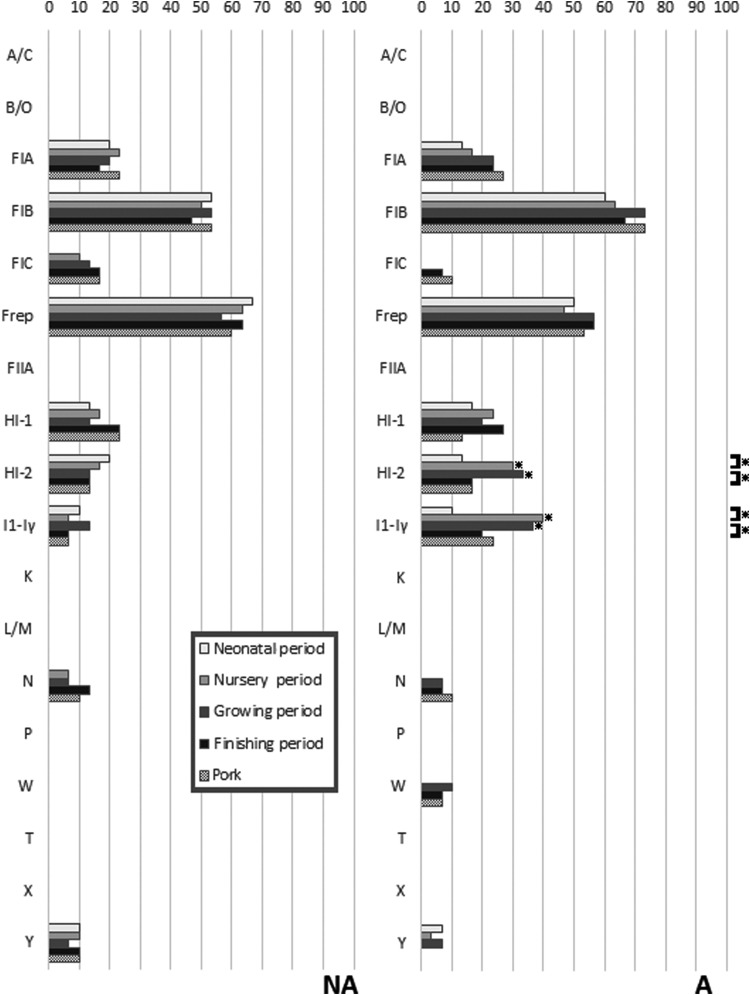
Differences in carriage of 18 plasmid replicons in *E. coli* from pigs in different periods of the production cycle and postslaughter (pork) between the farm using in-feed antibiotics (A) and the farm not using antibiotics (NA). *Indicates a significant difference using chi-squared analysis (*p* < 0.05). Comparisons are made between farms at each sampling period, and within farms at successive sampling periods.

The prevalence of 18 plasmid replicon types detected in the *E. coli* isolates is presented in Fig. 3. Replicons IncA/C, IncB/O, IncFIIA, IncK, IncL/M, IncP, IncT, and IncX were not detected. The IncFIB and IncFrep replicons were the only ones commonly found in both farms. All the other replicon types were found at a low to moderate prevalence in both farms. Statistically significant differences between sampling times were only found on farm A, and these were for IncHI-2 and IncI1-Iγ, which were increased in the isolates from the nursery and grower periods compared to other periods. These two replicons also were statistically significantly more frequently found in isolates from farm A than in isolates from farm NA in both the nursery and grower periods.

In the conjugation assay, *E. coli* PCU12-4 (positive for *bla*_CTX-M-1_ group with a single IncI1-Iγ replicon) transferred *bla*_CTX-M-1_ group with a frequency of 3.8 × 10^−5^, while *E. coli* PCU12-5 (positive for *bla*_CTX-M-1_ group with a single IncHI2 replicon) and *E. coli* PCU12-6 (positive for *bla*_CTX-M-9_ group with a single IncHI2 replicon) transferred the *bla*_CTX-M_ gene with frequencies of 4.1 × 10^−6^ and 5.6 × 10^−6^, respectively, confirming the location of the genes on these conjugative plasmids.

### Molecular genotypic characterizations

**FIG. 4.**
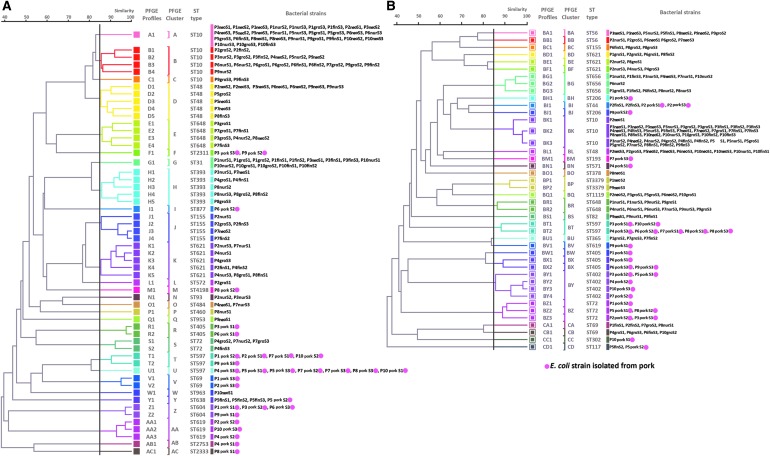
Genetic relatedness of *E. coli* strains analyzed by dendrograms generated based on PFGE data, with MLST results included (STs), divided according to farm type (farm NA with 55 PFGE types and farm A with 43 PFGE types). A round spot in *pink* indicates types that were found in pig meat. MLST, multilocus sequence typing; PFGE, pulsed-field gel electrophoresis. Genetic relatedness of *E. coli* strains analyzed by dendrograms generated based on pulsed field gel electrophoresis (PFGE) data, with multi-locus sequence typing (MLST) results included (STs), divided according to farm type [farm NA with 55 PFGE types **(A)** and farm A with 43 PFGE types **(B)**]. A round spot in *pink* indicates types that were found in pig meat.

**FIG. 5.**
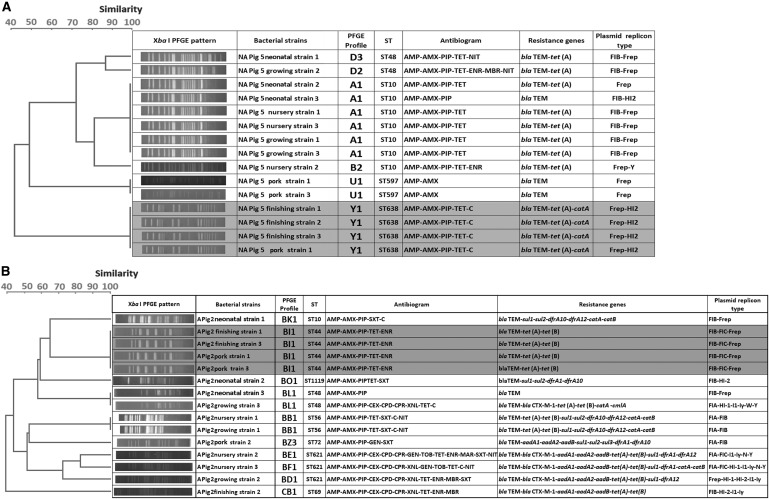

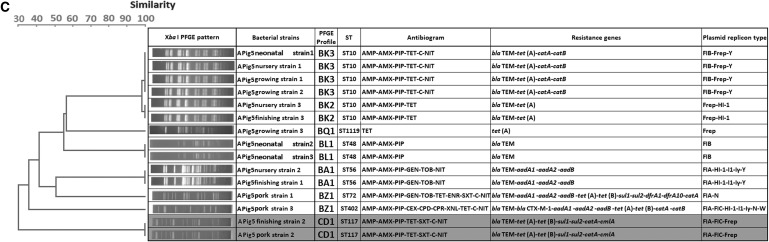
Datasets of *E. coli* isolates from three pigs showing the genetic relatedness, antibiogram, resistance gene profiles, and replicon profiles for isolates from live pigs and pork that share the same molecular type; **(A)**, isolates from pig number 5 on farm NA; **(B)**, isolates from pig number 2 on farm A; **(C)**, isolates from pig 5 on farm A. *Shaded rows* outline the isolates from pork and earlier samples from the live pig that shared a PFGE type.

The 300 isolates had diverse molecular types: those from farm A belonged to 25 STs and 43 PFGE types, while those from farm NA belonged to 24 STs and 55 PFGE types. A total of 41 STs were detected, and only nine were shared by isolates from the two farms. No PFGE types were shared between the two farms. Strain diversity (as assessed by comparing PFGE types using the Shannon diversity index) was higher on farm NA (*H*’ = 3.38) than on farm A (*H*’ = 3.31). PFGE typing was more discriminatory than MLST for identification of individual strains, but both methods gave broadly similar results in terms of depicting relationships between isolates (Fig. 4). In the case of three pigs, one or two isolates recovered from pork shared the same molecular types and resistance profiles with those recovered from the feces of the corresponding live animal at earlier periods: this occurred in one pig on farm NA (ST44) and two on farm A (ST638 and ST117) (Fig. 5 and Supplementary Table S1; Supplementary Data are available online at www.liebertpub.com/mdr). These shared strains did not show the ESBLP trait or aminoglycoside resistance. Sharing of strains with exactly the same characteristics between live pigs and pork was not found for the other 17 pigs.

For the 100 sampling activities, there were only 11 instances where the STs of the three isolates taken from the same pig were the same (Supplementary Table S1). Eight of these 11 instances were in pigs from farm NA and three from farm A. Nine of the incidences involved ST10, with the other two being ST638 and ST597. In 40 cases, two of the three isolates had the same ST (19 on farm NA and 21 on farm A), and in the other 49 cases, three different isolates were recovered.

**FIG. 6.**
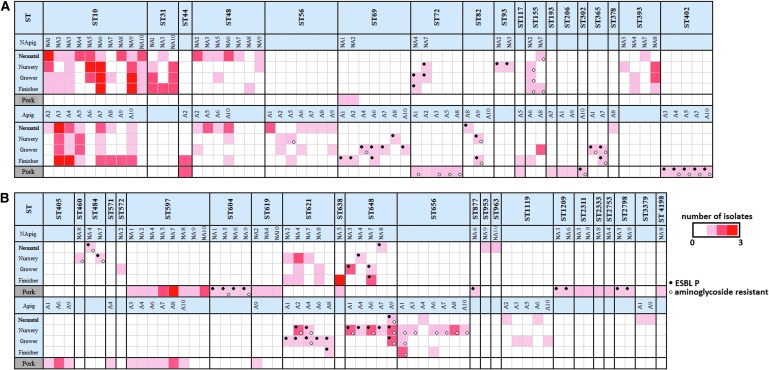
Longitudinal monitoring of the frequency of ST and distribution of ESBLP and aminoglycoside resistance among *E. coli* isolates from the two farms at different sampling periods. *Black circle* indicates the detection of ESBLP *E. coli*. *Light circle* indicates the detection of aminoglycoside resistance.

Figure 6 presents a synopsis of the relationships of *E. coli* isolates at each production period and in pork, based on their STs. Although ST10 was not found in pork, it was the most common type found in live animals from both farms (57/150 from farm NA and 40/150 from farm A). The difference in rates for ST10 between the two farms was significant (*p* < 0.05). In nearly every case, the isolates from pork belonged to different STs from those in the live pigs: as previously mentioned, exceptions were ST638 in farm NA, and ST44 and ST117 in farm A. Of the 12 STs that included isolates recovered from pork, six had isolates with ESBLP characteristics (ST302, ST402, ST604, ST877, ST1209, and ST2798). These isolates were only found in pork, and four of the STs were only isolated from the NA farm. ST597 did not show ESBLP characteristics, but it was of interest because although it was not found in live pigs, it was common in pork derived from pigs from both farms. STs containing isolates with ESBLP characteristics did occur in pigs on farm NA, but such strains were more common and sometimes also associated with aminoglycoside resistance in pigs on farm A.

## Discussion

Even though it is understood that commensal bacteria in animals may act as a reservoir of AMR, to date, evidence verifying a direct link between these organisms in livestock and their occurrence in meat products is limited.^22^ This study sought further evidence by undertaking longitudinal monitoring of AMR in commensal *E. coli* isolated from cohorts of pigs sampled at different stages of the pig production cycle, and comparing these to isolates from meat taken from the same animals after slaughter. Meat rather than feces was sampled postslaughter, as this is the product to which consumers are exposed. The design of the study was not able to account for any potential postslaughter cross-contamination of carcasses by *E. coli* strains during their handling and processing before sampling occurred.

The study examined susceptibility to large numbers of different antimicrobials, including consideration of ESBLP and aminoglycoside resistance because of the high significance of these traits for human bacterial infections. In this study, as in a previous study,^9^ aminoglycoside resistance rates and the presence of aminoglycoside resistance genes (*aadA1*, *aadA2*, and *aadB*) significantly increased, but only in the nursery and grower phases, and only on farm A. Hence the routine use of unrelated antimicrobials increased resistance to these critically important antimicrobials in growing animals.^9,23^ The reason(s) for this remain unclear, but may be due to some form of co-selection. Although AMR was common, and resistance to β-lactam group antibiotics and tetracycline were again found in most isolates, the resistance rates in isolates from fattening pigs for cefalexin, cefpirome, cefpodoxime, ceftiofur, chloramphenicol, enrofloxacin, gentamicin, imipenem, marbofloxacin, nitrofurantoin, tobramycin, trimethoprim/sulfamethoxazole, and ESBLP *E. coli* were lower than in a previous study in Thailand.^9^ This difference was presumably due to farm-specific factors, as the laboratory methods used in the two studies were the same.

To add another dimension to this study, two farms were followed, with one routinely using in-feed antimicrobials and the other not doing this. Previously we have shown that this routine in-feed antimicrobial use increases AMR rates in commensal *E. coli* in fattening pigs in Thailand,^9^ and hence we hypothesized that pork from farms using in-feed antimicrobials might be more heavily contaminated with AMR commensal bacteria following slaughter compared to farms not using antimicrobials. Nevertheless, in this study, no significant differences were found in AMR rates in isolates from pork from the two farms. Routine antimicrobial use on farm A only significantly increased resistance rates to aminoglycosides and the presence of ESBLP *E. coli*, and only in the nursery and grower periods.

In general, farm management and geographical origin have been shown to influence genetic diversity and the presence of AMR genes found in porcine *E. coli* isolates.^24^ In previous studies, a high clonal diversity of *E. coli* in pigs has been shown to occur both at the individual and pen levels,^25^ although individual strains may come to dominate. For example, in one recent cross-sectional study in a Danish pig farm not using antimicrobials, *E. coli* strains of ST10 and ST58 commonly were recovered throughout the growing period.^20^ In this study, three colonies from a high dilution (10^−4^) of each sample were selected for analysis to enhance the recruitment of a variety of *E. coli* strains.^9,26^ Using this sampling method, ST10 was the major type found in live animals in both farms, but it was not found in pork meat. ST10 *E. coli* also has been reported in humans, chickens, and other animals.^27,28^ In contrast to ST10, ST597 was the most common type found in pork from both farms, but it was not identified in live animals. It also was not ESBLP or resistant to aminoglycosides. ST597 could have been a cross-contaminant from the environment of the abattoir, from equipment, or from other carcasses, although the pigs from the two farms were killed in different abattoirs and so no single external source existed. ST597 has been reported as an enteric foodborne pathogen in humans,^29^ so identifying the route for its appearance in pig meat in abattoirs is important. Further studies into transmission of *E. coli* with AMR characteristics in the food chain are required: in particular, Hazard Analysis and Critical Control Points should be applied during farm to abattoir transportation, and through the standard slaughtering process, meat trimming, and packaging. The dominant *E. coli* types found in meat may have attributes that could help to explain why they are present rather than other types found in live animals.

As anticipated, molecular tracing and evaluation of strain relationships using MLST and PFGE gave similar results.^30^ Most *E. coli* types in pork were not detected in live pigs, except for STs 44, 117, 155, and 638 that included non-ESBPL *E. coli* and were negative for *bla*_CTX-M_ genes. As with previous cross-sectional surveys, no relationship was found between molecular types that contained genes encoding ESBLP and aminoglycoside resistance on the farm and their presence in the abattoir.^31^

Resistance to amino-penicillins (ampicillin and amoxicillin) and urevido-penicillin (piperacillin) conferred by the *bla*_TEM_ gene and tetracycline resistance conferred by the *tet*(A) gene were the most common forms of AMR in both farms at all observation periods, and was consistent with results of previous cross-sectional studies in Southeast Asia.^32^ The persistence of *bla*_TEM_ and *tet*(A) genes in *E. coli* may not be caused by direct selective pressure, but may imply an abundance of *bla*_TEM_ and *tet*(A) genes in Enterobacteriaceae bacteria in the region.^33^ Despite cephalosporins and aminoglycosides not being used on the farms, ESBLP *E. coli* containing *bla*_CTX-M-1 group_ and aminoglycoside-resistant *E. coli* containing *aadA1*, *aadA2*, and *aadB* were common in the nursery period in farm A. This confirmed the findings of our previous study^9^ and might be explained by a co-harboring of multiresistance genes, including *cfr* and *bla*_CTX-M_ genes, on a conjugative plasmid.^34^

Replicons IncFrep and IncFIB are reported as the most common types found in *E. coli* from humans and animals, and this was seen in both groups in this study. These plasmids, which encode factors involved in iron uptake, toxin production, enzymes, and a variety of resistance genes, for example, *bla*_CTX-M_, are widely spread in Enterobacteriaceae.^35^ In farm A, the high frequency of IncI1-Iγ, IncHI2, in *E. coli* in the nursery period was strongly correlated with the detection of ESBLP and the *bla*_CTX-M-1_ group gene. In previous studies, IncHI2 was found to carry not only the *bla*_CTX-M -2_ gene but also a variety of genes encoding sulfonamide, aminoglycoside, tetracycline, and streptomycin resistance.^36^ IncI1-Iγ carrying *bla*_CTX-M-1_ is also the most common plasmid found in *E. coli* from livestock.^37^ Thus, the nursery and grower pigs from farm A could act as an important reservoir of AMR on the farm, and possibly have an impact on public health by amplifying resistance genes that eventually make their way to other zoonotic pathogens or to the environment.

In conclusion, commensal *E. coli* with AMR traits (especially resistance to β-lactam group antibiotics and tetracycline) were common in pigs from both farms at all sampling periods. *E. coli* that were aminoglycoside resistant and ESBLP were commonly found in the nursery and grower periods, and were significantly more common in the farm that routinely used unrelated classes of antibiotics. Molecular typing showed that on both farms, strains from pigs and pork were different in terms of their clonal types and characteristics. This suggests that on-farm resistance, which is encouraged by antimicrobial usage, does not necessarily reflect the attributes of *E. coli* found in meat at the abattoir. Further work is required to identify potential sources of resistant *E. coli* found in pig meat following slaughter.

## Supplementary Material

Supplemental data
