# Supplementary material for: Antimicrobial Resistance in Commensal Escherichia coli Isolated from Pigs and Pork Derived from Farms Either Routinely Using or Not Using In-Feed Antimicrobials
Source: Microb Drug Resist. 2018 Sep 10;24(7):1054–66. doi: 10.1089/mdr.2018.0154 (PMC6154756; doi:10.1089/mdr.2018.0154)
Supplement: Supplemental data [file Supp_Table1.pdf]

Supplementary Data

Supplementary Table S1. Sequence Types of Individual *E. coli* Isolates from Each Pig at Each Sampling Period on Farms A and NA

	Neonatal period	*Nursery period*	*Growing period*	*Finishing period*	*Pork*
NA1 strain 1	ST10	ST31	ST31	ST31	ST604
NA1 strain 2	ST10	ST10	ST31	ST31	ST597
NA1 strain 3	ST10	ST10	ST10	ST10	ST69
NA2 strain 1	ST10	ST155	ST72	ST621	ST597
NA2 strain 2	ST48	ST93	ST10	ST10	ST619
NA2 strain 3	ST48	ST621	ST155	ST155	ST69
NA3 strain 1	ST31	ST393	ST648	ST31	ST405
NA3 strain 2	ST10	ST10	ST10	ST10	ST604
NA3 strain 3	ST48	ST93	ST648	ST31	ST2311
NA4 strain 1	ST484	ST621	ST393	ST393	ST2753
NA4 strain 2	ST10	ST648	ST72	ST621	ST619
NA4 strain 3	ST10	ST621	ST621	ST72	ST597
NA5 strain 1	ST48	ST10	ST10	ST638	ST597
NA5 strain 2	ST10	ST10	ST48	ST638	ST638
NA5 strain 3	ST10	ST10	ST10	ST638	ST597
NA6 strain 1	ST48	ST10	ST10	ST10	ST405
NA6 strain 2	ST48	ST10	ST10	ST10	ST877
NA6 strain 3	ST10	ST10	ST10	ST10	ST604
NA7 strain 1	ST393	ST621	ST648	ST648	ST597
NA7 strain 2	ST155	ST72	ST72	ST155	ST597
NA7 strain 3	ST48	ST484	ST72	ST648	ST597
NA8 strain 1	ST10	ST460	ST621	ST621	ST2333
NA8 strain 2	ST648	ST393	ST393	ST393	ST4198
NA8 strain 3	ST10	ST393	ST393	ST48	ST597
NA9 strain 1	ST953	ST10	ST10	ST10	ST604
NA9 strain 2	ST10	ST10	ST10	ST10	ST2311
NA9 strain 3	ST48	ST48	ST10	ST10	ST597
NA10 strain 1	ST963	ST31	ST31	ST31	ST597
NA10 strain 2	ST10	ST31	ST31	ST31	ST597
NA10 strain 3	ST10	ST10	ST10	ST10	ST619
A1 strain 1	ST56	ST648	ST31	ST69	ST405
A1 strain 2	ST3379	ST656	ST365	ST656	ST72
A1 strain 3	ST56	ST648	ST656	ST656	ST206
A2 strain 1	ST10	ST56	ST56	ST44	ST44
A2 strain 2	ST1119	ST621	ST621	ST69	ST72
A2 strain 3	ST48	ST621	ST48	ST44	ST44
A3 strain 1	ST10	ST10	ST1119	ST10	ST597
A3 strain 2	ST10	ST10	ST10	ST10	ST402
A3 strain 3	ST10	ST656	ST10	ST10	ST72
A4 strain 1	ST10	ST648	ST69	ST10	ST571
A4 strain 2	ST10	ST10	ST10	ST10	ST402
A4 strain 3	ST656	ST621	ST621	ST10	ST597
A5 strain 1	ST10	ST10	ST10	ST56	ST72
A5 strain 2	ST48	ST56	ST10	ST117	ST117
A5 strain 3	ST48	ST10	ST1119	ST10	ST402
A6 strain 1	ST56	ST648	ST621	ST155	ST405
A6 strain 2	ST1119	ST656	ST56	ST656	ST597
A6 strain 3	ST48	ST648	ST69	ST69	ST405
A7 strain 1	ST10	ST656	ST10	ST10	ST597
A7 strain 2	ST10	ST10	ST69	ST365	ST402
A7 strain 3	ST56	ST648	ST365	ST10	ST193
A8 strain 1	ST378	ST69	ST621	ST10	ST597
A8 strain 2	ST56	ST656	ST155	ST621	ST72
A8 strain 3	ST10	ST656	ST155	ST10	ST597
A9 strain 1	ST82	ST82	ST648	ST82	ST619
A9 strain 2	ST56	ST648	ST56	ST10	ST206
A9 strain 3	ST3379	ST648	ST648	ST10	ST405
A10 strain 1	ST48	ST48	ST1119	ST48	ST302
A10 strain 2	ST10	ST656	ST69	ST10	ST597
A10 strain 3	ST48	ST10	ST10	ST10	ST402
